# Soil Microbial Biomass and Fungi Reduced With Canola Introduced Into Long-Term Monoculture Wheat Rotations

**DOI:** 10.3389/fmicb.2019.01488

**Published:** 2019-07-11

**Authors:** Jeremy C. Hansen, William F. Schillinger, Tarah S. Sullivan, Timothy C. Paulitz

**Affiliations:** ^1^ Northwest Sustainable Agroecosystems Research Unit, USDA-Agricultural Research Service, Washington State University, Pullman, WA, United States; ^2^ Department of Crop and Soil Sciences, Washington State University, Pullman, WA, United States; ^3^ Wheat Health, Genetics, and Quality Research Unit, USDA-Agricultural Research Service, Washington State University, Pullman, WA, United States

**Keywords:** *Brassica napus* L, crop rotation, soil microbial communities, phospholipid fatty acid analysis, wheat

## Abstract

With increasing canola (*Brassica napus* L.) acreage in the Inland Pacific Northwest of the USA, we investigated the effect of this relatively new rotational crop on soil microbial communities and the performance of subsequent wheat (*Triticum aestivum* L.) crops. In a 6-year on-farm canola-wheat rotation study conducted near Davenport, WA, grain yields of spring wheat (SW) following winter canola (WC) were reduced an average of 17% compared to SW yields following winter wheat (WW). Using soil samples collected and analyzed every year from that study, the objective of this research was to determine the differences and similarities in the soil microbial communities associated with WC and WW, and if those differences were associated with SW yield response. Microbial biomass and community composition were determined using phospholipid fatty acid analysis (PLFA). The WC-associated microbial community contained significantly less fungi, mycorrhizae, and total microbial biomass than WW. Additionally, reduced fungal and mycorrhizal abundance in SW following WC suggests that the canola rotation effect can persist. A biocidal secondary metabolite of canola, isothiocyanate, may be a potential mechanism mediating the decline in soil microbial biomass. These results demonstrate the relationship between soil microbial community composition and crop productivity. Our data suggest that WC can have significant effects on soil microbial communities that ultimately drive microbially mediated soil processes.

## Introduction

Soil microorganisms are an integral part of many soil processes in agroecosystems. The soil microbial community plays a key role in aggregate stability (Duchicela et al., 2013; Graf and Frei, 2013), mineralization and stabilization of organic matter (Lützow et al., 2006; Schmidt et al., 2011), nutrient cycling (Kennedy and Papendick, 1995; Talbot et al., 2013), soil ecological function, and biological stability (Griffiths and Philippot, 2013). These processes can be altered by shifts in the activity and composition of microbial communities caused by environmental conditions or agricultural practices. Soil microbial communities are sensitive to changes in tillage (Larkin and Honeycutt, 2006), cover cropping (Pritchett et al., 2010), soil amendments (Marschner et al., 2003; Tian et al., 2015), soil moisture (Kennedy and Schillinger, 2006; Brockett et al., 2012), and temperature (Petersen and Klug, 1994; Bossio et al., 1998). However, no single agricultural input or management strategy imposed on a soil determines the composition of the microbial community. Rather, a combination of edaphic and dynamic factors, including crop rotation, residue management, soil type, tillage, and climate, all interact to influence the microbial community (Bünemann et al., 2008; Gil et al., 2011; Zhang et al., 2014).

Agroecosystems with generally well-defined histories often have controlled inputs that directly influence soil microbial communities (Minoshima et al., 2007; Buyer et al., 2010). In soils with such histories, above-ground changes in crop species (Grayston et al., 1998; Marschner et al., 2001; Ladygina and Hedlund, 2010), crop rotation sequence (Alvey et al., 2003; Bünemann et al., 2008; Schillinger et al., 2010; Xuan et al., 2012), and tillage (Schillinger et al., 2007; Gil et al., 2011; Zhang et al., 2014) will influence below-ground physical and biological properties. Agricultural soils of the dryland cropping regions of the Pacific Northwest have historically been used to produce wheat in 2- or 3-year rotations (Schillinger et al., 2006).

Crop rotation has been well documented as a driving factor influencing soil microbial community composition (Drijber et al., 2000; Larkin and Honeycutt, 2006; Xuan et al., 2012; Valetti et al., 2016). Soil fungal (Zhang et al., 2014; Valetti et al., 2016) and bacterial communities are influenced by crop rotation (Alvey et al., 2003; Larkin and Honeycutt, 2006; Xuan et al., 2012) and will vary with different crops in a rotation sequence (Grayston et al., 1998; O’Donnell et al., 2001). An increase in the frequency of oilseed rape (*Brassica napus*) in wheat-oilseed-based rotations is reported to significantly affect soil fungal communities (Hilton et al., 2013). Reduced diversity among soil fungal and rhizosphere bacterial communities associated with *Brassica* plants has also been observed (Bennett et al., 2014).

Knowledge of how plant-derived phytochemicals influence soil microbiota through positive or negative root-soil interactions is increasing (Bais et al., 2006; Kong et al., 2008; Lorenzo et al., 2013). Phytochemicals released in root exudates and through decomposition of residues have enormous potential to affect soil microbial community composition and structure (Dong et al., 2014). Glucosinolates (GSLs) and isothiocyanates (ITCs) of *Brassica* plant species are phytochemicals of interest due to their biofumigation potential (Brown and Morra, 1997; Rumberger and Marschner, 2003; Matthiessen and Kirkegaard, 2006). The preferred method of biofumigation for soil-borne pathogen suppression is through incorporation of *Brassica* green manure crops or seed meal (Brown and Morra, 1997; Morra and Kirkegaard, 2002; Matthiessen and Kirkegaard, 2006; Mazzola et al., 2015). Biocidal compounds (ITCs) of *Brassica* seed meal drastically reduced soil fungal populations, while ITC amendments caused a shift in the fungal community composition (Hu et al., 2015). As a rotational crop, however, the seed is harvested and the residue material is left on the soil surface, rather than incorporated. Therefore, any influence on soil microbial communities relies on GSLs, ITCs, or other compounds released through leaf washings (Walsh et al., 2014), root exudates (Marschner et al., 2001), or detached root material (Bennett et al., 2014).

The GSL gluconasturtiin of canola roots (Sarwar et al., 1998) hydrolyzes to form 2-phenylethylisothiocyanate and is released by living canola roots into the surrounding soil (Rumberger and Marschner, 2003). Root GSL and ITC concentration is elevated compared to the above-ground leaf and shoot material and is in direct contact with rhizosphere microorganisms (van Dam et al., 2009). Exudation of ITC from canola roots is sufficient to effectively suppress some soil-borne pathogens (Haramoto and Gallandt, 2004) and reduce specific soil microorganisms within the active portion of the soil microbial community (Smith and Kirkegaard, 2002; Rumberger and Marschner, 2003). Recently, Lay et al. (2018) observed significant differences in the rhizosphere microbiome of canola compared to wheat. Changes in the soil microbial community composition of wheat have been attributed to the influence of the preceding oilseed rape crop (Hilton et al., 2018). Therefore, canola as a rotational crop could induce changes in the soil microbial community composition (Smith et al., 2004; Valetti et al., 2016; Hilton et al., 2018).

Critical microbial processes that could be disrupted by exposure to the hydrolysis products of GSLs include nitrification, nitrogen fixation, and plant-microbial symbiotic associations (Haramoto and Gallandt, 2004). Free-living nitrogen-fixing bacteria populations were reported to be lower following a canola crop (Kirkegaard and Sarwar, 1999). Total abundance of ammonium-oxidizing and nitrite-oxidizing bacteria populations were reduced and nitrification capacity decreased by 35–65% after exposure to ITCs, while 2-phenylethylisothiocyanate completely inhibited nitrification (Bending and Lincoln, 2000). Abundance of arbuscular mycorrhizae (AM fungi) declined by approximately 60% after a 28-day exposure to rape seed extract ITC (Siebers et al., 2018), and mycorrhizal hyphae were negatively influenced by *Brassica* root exudates (Koide and Schneider, 1992). Reduced wheat yields in plots previously planted to canola were attributed to poor colonization with AM fungi, indicated by a significant correlation between yield of wheat and the levels of AM fungi (Owen et al., 2010).

In a 6-year on-farm rotation study conducted near Davenport, WA, yield data were collected for both WC and WW at harvest of plots in the first year of the crop sequence. In the second year of the crop sequence, yield data were collected for SW following both WC and WW. Schillinger and Paulitz (2018) reported that the average yield for WC and WW was 2,603 and 6,375 kg ha^−1^, respectively. Schillinger and Paulitz (2018) further reported that average SW grain yield following WC and WW was 3,267 and 3,932 kg ha^−1^, respectively. Spring wheat yields were significantly greater after WW compared to WC in 3 of the 5 years. The 5-year average yield for SW after WC was significantly (*p* < 0.0001) reduced by 17% compared with SW after WW. Averaged over the years, there were no differences in soil water use or soil water content between WC and WW at time of planting of SW (Schillinger and Paulitz, 2018). There were no visible or measurable foliar or root diseases in any crop in any year, nor were any root lesion nematodes detected (Schillinger and Paulitz, 2018). All plots were essentially weed-free every year. All plots were adequately fertilized. Schillinger and Paulitz (2018) concluded that unidentified factors caused the significant 17% yield reduction of SW following WC vs. WW and suspected this difference could be related to soil microbiology. This generated the interest and focus of the current study for an in-depth microbiological analysis of the soil cores collected and archived every year from the study.

Phospholipid fatty acid (PLFA) analysis is frequently used to assess the composition of microbial communities in agricultural soils (Finney et al., 2017). Microbial community PLFA analysis provides sensitive, reproducible measurements to explore the soil microbial community resulting in estimates of both microbial community composition and biomass (Kaur et al., 2005). Bacterial and fungal cell walls are composed of phospholipids that rapidly degraded after cell death and therefore represent a reliable *in situ*, quantitative measure for living microorganisms in soil (Kaur et al., 2005; Pollierer et al., 2015). Data from phospholipid analysis represent the viable microbial biomass, whereas DNA sequencing techniques may include relic DNA from deceased microbial cells that have been exposed to ITC (Siebers et al., 2018). While other molecular methods can deliver a more detailed description of microbial diversity, PLFA offers the advantage of quantifying the total microbial biomass at a lower cost (Finney et al., 2017). When comparing PLFA profiling to 16S rRNA gene metabarcoding, Orwin et al. (2018) determined the two techniques to give broadly comparable results for detecting changes in community composition and ecosystem functions. PLFA profiling is a powerful tool to monitor changes in microbial communities (Buyer and Sasser, 2012; Zhang et al., 2014) and is widely used to investigate microbial community dynamics during land-use change (Smith et al., 2015).

The objective of our study was to examine the short-term influence of canola as a rotational crop on soil microbial communities that have developed under traditional wheat monoculture rotations. The study was initiated on land that had been in monoculture cereal production for 140 years and managed using direct seeding (i.e., no-till) for 15 years in a 3-year WW-SW-NTF (no-till summer fallow) rotation. Canola or any other broadleaf crop had never been previously grown on this land. Long histories and consistent rotations allow for a pseudo isolation of variables and the ability to focus on the effects of canola on subsequent wheat crops. We hypothesized that the canola-associated soil microbial community would be differentially influenced by exposure to residues of canola plants and particularly exudates of canola roots. More specifically, we hypothesized that exposure to residues of winter canola plants and particularly exudates of canola roots will shift the soil microbial community, compared to a previous rotation of winter wheat. PLFA analysis coupled with microbial enzyme activities could increase the current knowledge of the presence of canola-associated exudates and residues in soil and the relative impact on the corresponding soil microbial communities.

## Materials and Methods

### Site Description and Experimental Design

A 6-year on-farm crop rotation experiment was conducted during the 2008–2013 crop years at the Hal Johnson farm (47°40′16.86″N 118°1′58.87″W) located 9 km east of Davenport, WA. Long-term annual precipitation at the site averages 432 mm. Crop-year (September 1 to August 31) precipitation during the study period ranged from 342 to 510 mm and averaged 396 mm during the 6-year experiment.

The soil is a Hanning silt loam (fine-silty, mixed, superactive, and mesic pachic argixerolls) with 0–7% slopes and a depth of 1.5–2 m to restrictive layers (NRCS, 2018). The experimental crop rotations were WC-SW-NTF compared with the traditional WW-SW-NTF. This resulted in four distinct treatment conditions for our study: (1) WC, (2) WW, (3) SW following WC (SW-WC), and (4) SW following WW (SW-WW). In the 140-year farming history of this land, only cereal crops have been grown (i.e., no *Brassica* or other broadleaf crops). Since the WC and WW were established on different land parcels each year, the WC was always planted into “virgin” canola soil. All crops were direct-seeded (without tillage, to maintain the no-till management) into the standing stubble of the previous crop with a hoe-opener drill with 10 cm paired rows on 30 cm row spacing. The drill was equipped to apply liquid fertilizer in a deep band between the paired rows.

Experimental design was a randomized complete block with six replications. Individual plot size was 30 m × 5 m. Seeding rate for the three crops varied slightly each year and averaged 6, 83, and 100 kg ha^−1^ for WC, WW, and SW, respectively. Fertilizer application rates of nitrogen (N), phosphorous (P), and sulfur (S) were based on soil test results. Winter canola and WW have similar N and P requirements, but WC has a greater S requirement than WW (Koenig, 2005). Therefore, the S fertilizer applied each year was based on the needs of WC. Both WC and WW always received identical rates of N, P, and S fertilizer. Soils in the region contain naturally high quantities of potassium (K) and that nutrient was not applied. Fertilizer was applied in several different combinations over the years by coulter injection in late summer and in the spring as well as at time of planting of WC, WW, and SW.

Winter canola and WW were planted prior to September 15, and SW was planted in late April-early May. Details of seeding rates, cultivars, plant stands, fertilizers, and herbicides used throughout the experiment are provided in Schillinger and Paulitz (2018). Crops in all plots were maintained essentially weed-free. Winter canola was lost to freezing damage in 2010; thus, the experiment was abandoned that year. Since SW follows winter crops in this rotation, no SW data could be collected in 2011.

### Soil Sampling

Soil samples for microbial analyses were collected from 0 to 5, 5 to 10, and 10 to 15 cm depths to account for stratified soil properties often produced in long-term no-till systems. Soil was sampled from all plots in mid-May, when WW was tillering, WC was at inflorescence emergence, and SW seedlings were newly emerged. From each replicated plot, seven 3.0-cm-diameter soil cores at each depth were combined to form a composite sample. The seven cores were collected randomly across the length and width of each plot, avoiding borders. Sample collection occurred each year at the same time from 2009 through 2015. Samples collected in the first year of the rotation were from WC and WW. Samples from the subsequent year were collected from SW following WC (SW-WC), and SW following WW (SW-WW) resulting in four treatment combinations. After collection, samples were immediately transported on ice in the dark to the USDA laboratory located in Pullman, WA. Subsamples of the composite soil were collected in sterile tubes and stored at −80°C until analysis.

### Soil Chemical Analyses

Soil pH and electrical conductivity (EC) were determined as outlined in McLean (1982). A soil slurry of 1:1 soil to distilled, deionized water was prepared and allowed to mix overnight by end-over-end shaking at 40 rpm. After shaking, the slurry was then centrifuged at 2,831 × *g*, and the resulting supernatant was measured. Soil solution pH was measured with an Orion Research 811 (Boston, MA) pH meter, and EC was measured using a digital conductivity meter (VWR International, Bristol, CT).

### Soil Microbial Enzyme Activity

As indicators of microbial activity, soil β-glucosidase (BGA) and dehydrogenase (DEA) were determined as described in Tabatabai and Dick (2002). Colorimetric measurement, and calculation of p-nitrophenol (PNP) and 2,3,5-triphenyl formazan (TPF) are described in Hansen et al. (2018). Briefly, colorimetric measurements of extracted solutions were completed on a BioTek microplate reader (BioTek, Winooski, VT), set to a wavelength of 410 nm (BGA), and 492 nm (DEA). Results for BGA are expressed in micrograms of p-nitrophenol (PNP) released per gram of dry soil per hour, while DEA is expressed as micrograms of 2,3,5-triphenyl formazan (TPF) per gram of dry soil per hour.

### Soil Microbial Biomass and Community Composition

The protocol described by Buyer et al. (2010) was followed for PLFA extraction. Lyophilized soil (2.5 g) was extracted according to Bligh-Dyer extraction (Bligh and Dyer, 1959), with 12 ml of extractant as described by Petersen and Klug (1994). Lipid separation was completed with a solid-phase extraction column. Phospholipids were eluted with 5 ml of methanol. After evaporation under N_2_, the phospholipids were transesterified to fatty acid methyl esters, extracted into 4 ml of hexane, and evaporated. Phospholipids were analyzed on an Agilent 6890 GC equipped with autosampler, split-splitless injector, Agilent Ultra 2 column, and flame ionization detector. The GC was controlled with Agilent Chemstation and MIDI Sherlock software (Microbial ID, Inc., Newark, DE, USA). Hydrogen was used as the carrier gas at 66.9 kPa constant pressure. The split ratio of the MIDI Eukaryote method was changed from 1:100 to 1:50 to increase sensitivity. The initial oven temperature was 170°C and programmed to ramp at 5°C min^−1^, with a final hold of 12 min at 300°C. An internal standard of 19:0 nonadecanoic acid methyl ester was used for quantification. Peak chromatographic responses for fatty acids were converted to molarity by reference to the internal standard 19:0 Methyl nonadecanoate (Sigma-Aldrich Inc., St. Louis, MO, USA). Peak areas of carbon chain lengths between 12:0 and 20:0 were summed into biomarker groups (Hansen et al., 2018). Iso and anteiso branched fatty acids were used as biomarkers for Gram-positive (Gram+) bacteria (Zelles, 1999). Gram-negative bacteria (Gram−) were identified by monounsaturated fatty acids, and cyclopropyl 17:0 and 19:0 (Zelles, 1997). The fatty acids 18:2 ω6c and 18:1 ω9 were used as biomarkers for fungi (Vestal and White, 1989; Frostegård and Bååth, 1996; Bååth, 2003). Arbuscular mycorrhizae (AM fungi) biomarker included 16:1 ω5c (Olsson, 1999) and 20:1 ω9c (Madan et al., 2002). Total of all PLFA biomarkers (T-PLFA) was considered the viable microbial biomass.

### Statistical Analysis

Analysis of variance (ANOVA) was conducted for soil chemical, enzyme activity, and microbial biomarker data using a randomized complete block design with a one-way treatment structure ANOVA. Differences were assessed using linear mixed models (PROC MIXED SAS version 9.4; SAS Inc., Cary, NC) with treatment as the fixed-effect factor and year as the random-effect factor. To determine significant differences between treatment means, comparisons were performed through the GLMIMIX procedure at *p* ≤ 0.05. When *p*’s indicated significance, differences in treatment means were determined by Fisher’s protected LSD. Multivariate discriminant function analysis using the linear common covariance method (JMP, 1989–2007) was used to explore the primary effect of the crop treatment on the structure of the soil microbial community. This analysis maximizes the among-group variation relative to the within-group variation to identify differences between previously established groups (Lazcano et al., 2013). Linear discriminant analysis uses canonical plots to display the multivariate means of each group in the two dimensions that best differentiate the microbial communities. Bi-plots of the first two canonical variables illustrate the two dimensions that create maximum separation of microbial communities associated with each crop treatment. For clarity, the group means are shown and not individual points (*n* = 120). Each point is accompanied by a mean ellipse at the 95% confidence interval. By definition, two groups are significantly different from each other when the ellipses do not intersect (JMP, 1989–2007). The bi-plot vectors represent covariates (biomarker groups), with the length and direction of each vector proportional to the degree of association with the first two canonical variates. For all bi-plot presented in this paper, the first canonical variate (CV) accounts for the bulk the variance. Therefore, separations along the horizontal axis are more significant than those occurring along the vertical axis. Analysis was performed using the JMP software program (JMP, 1989–2007) at the *p* ≤ 0.05 significance level.

## Results

### Soil Properties and Enzyme Activities

**Figure 1:**
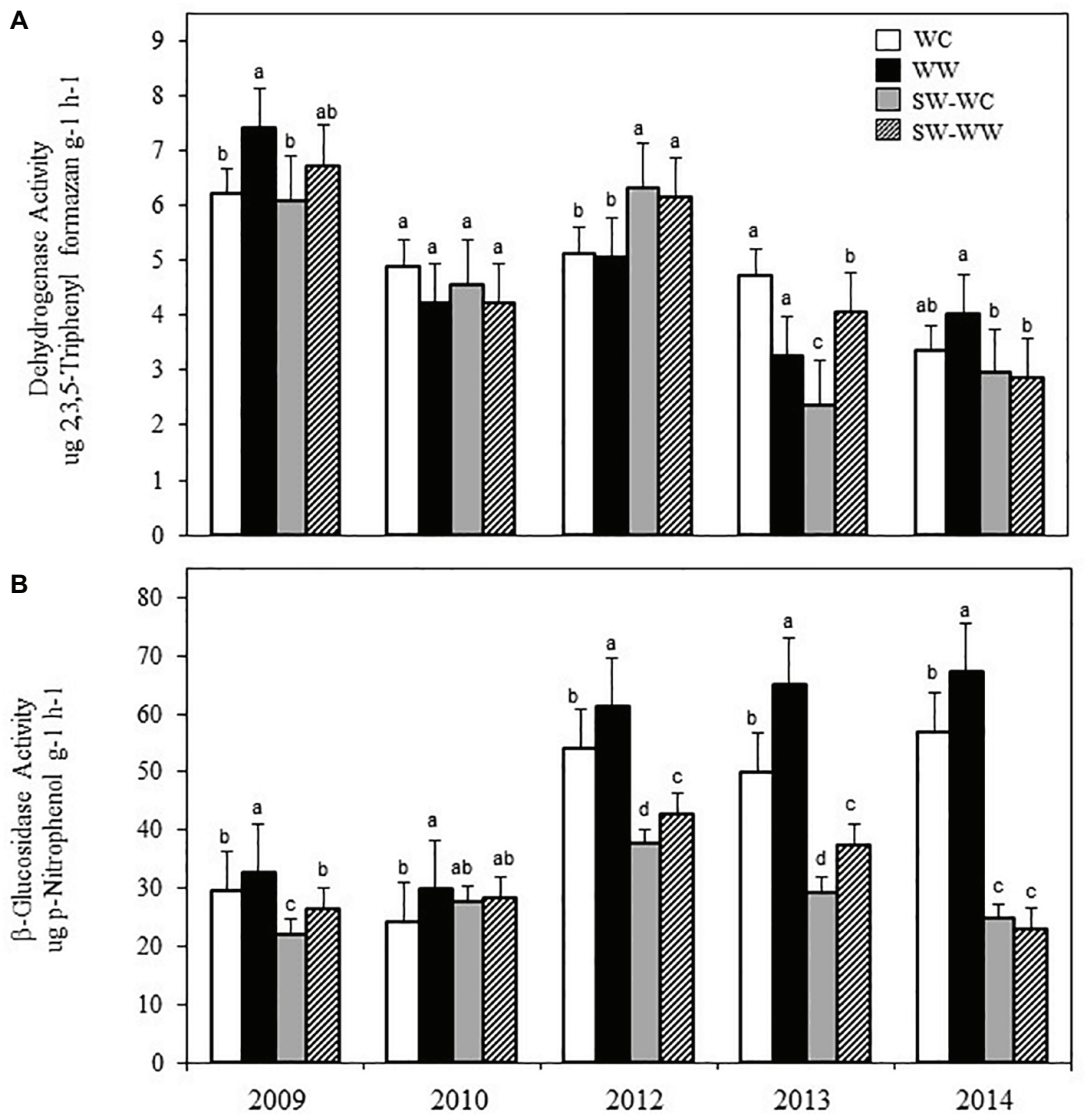
Dehydrogenase activity **(A)**, and β-glucosidase activity **(B)**, under different crop treatments from 2009 to 2014 at 0–5 cm. Error bars represent standard error. Different letters within years indicate significant differences at *p* < 0.05.

Crop rotation did not have a significant effect on the soil pH, EC, or gravimetric water content, which averaged 5.30 (±0.11) and 5.33 (±0.14), 97.90 (±1.97), and 96.59 (±1.49) μS cm^−1^, and 0.20 ± 0.09 and 0.21 ± 0.11 kg kg^−1^ for the WC and WW, respectively. Activity levels of DEA did not exhibit a consistent pattern of significant differences throughout the experiment (Figure 1A). Differences were observed in 2008 between WC and WW, and 2013 between SW-WW and SW-WC at 0–5 cm. Data for BGA did, however, exhibit significant differences between treatments at 0–5 cm (Figure 1B). In the winter-crop cycle of the crop sequence, BGA was greater in WW than in WC in all five crop years. For the ensuing SW, BGA was significantly greater in SW-WW vs. SW-WC in three of the five crop years. Consistent differences in enzyme activity at the 5–10 and 10–15 cm depths were not observed (data not shown).

### Soil Microbial Biomass and Community Composition

The absolute abundance of microbial lipid biomarkers in the 0–5 cm depth for fungi, AM fungi, and total viable microbial biomass (T-PLFA) were significantly greater in WW compared to WC when averaged across the duration of the experiment (Table 1). Similar decreases in biomarkers associated with SW-WC were detected in the second year of the rotation. Both fungi and AM fungi were significantly less in SW-WC compared to SW-WW (Table 1). Likewise, abundance of biomarkers was greater in WW compared to WC at the 5–10 cm depth in the first year of the rotation (Table 1). Significant reductions in fungi, AM fungi, and TPLFA were seen in WC at this depth. However, bacteria did not differ (Table 1). Differences in individual lipid biomarkers and T-PLFA were not observed at the 10–15 cm depth in either the first year WC and WW treatments, or the following SW-WC and SW-WW treatments (Table 1).

**Table 1:** Absolute abundance (nmol/g) of microbial lipid groups at 0–5, 5–10, and 10–15 cm depths from crop years 2009–2014.

Depth	Treatment	Fungi	AM fungi	Gram−	Gram+	Total
0–5 cm	WC	***1.11*** B	***0.22*** B	***1.68*** B	3.01 AB	***12.47*** B
	WW	***1.74*** A	***0.41*** A	***3.96*** A	3.66 A	***16.67*** A
	SW-WC	***0.98*** B	***0.18*** B	1.18 B	2.69 B	11.32 B
	SW-WW	***1.60*** A	***0.34*** A	1.32 B	3.12 AB	13.32 B
5–10 cm	WC	***0.52*** B	***0.10*** B	0.84 AB	1.56 AB	***8.55*** B
	WW	***0.80*** A	***0.18*** A	1.05 A	1.83 A	***9.95*** A
	SW-WC	***0.36*** C	***0.04*** C	0.62 BC	1.36 B	8.27 B
	SW-WW	***0.51*** D	***0.12*** D	0.55 C	1.25 B	7.76 B
10–15 cm	WC	0.49 A	0.08 A	0.93 A	1.60 A	3.94 A
	WW	0.49 A	0.09 A	0.83 A	1.59 A	3.77 A
	SW-WC	0.54 A	0.07 A	0.79 A	1.46 A	3.75 A
	SW-WW	0.59 A	0.08 A	0.83 A	1.53 A	3.84 A

Values are least square means (n = 120) across all years. Means within a column at each depth with different letters are significantly different (p ≤ 0.05). Bold italics indicate significant differences between WC and WW, or SW-WC and SW-WW.

**Figure 2:**
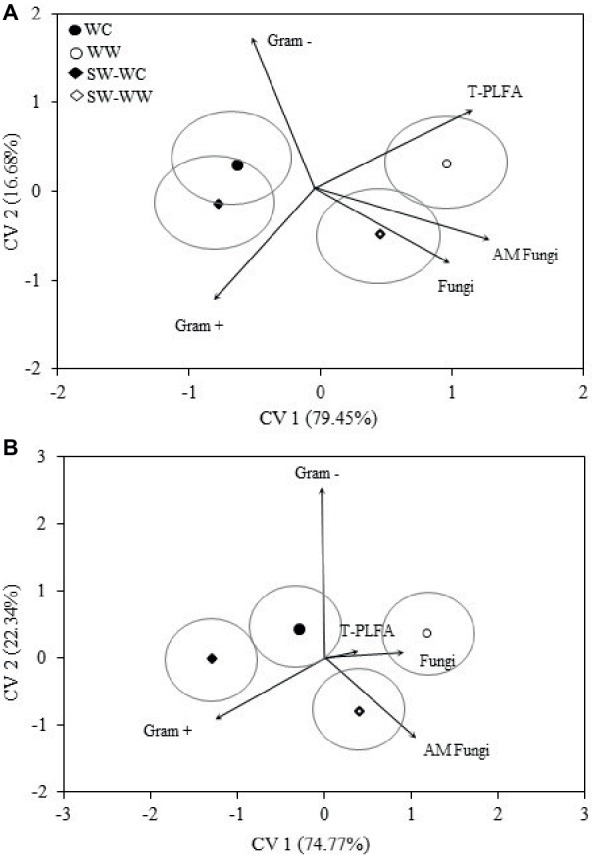
Canonical variates for lipid biomarker groups. Biomarker groups and total PLFA (T-PLFA) for 0–5 **(A)** and 5–10 **(B)** cm depths from crop years 2009 to 2014. Vectors represent standardized canonical coefficients and indicate the contribution of each biomarker group to each canonical variate. Each point represents the group centroid mean and is accompanied by a mean ellipse at the 95% confidence interval (treatments groups that differ significantly have confidence ellipses that do not intersect).

Discriminant analysis for the microbial biomarkers averaged across all years is represented as bi-plots in Figure 2. For both the 0–5 and 5–10 cm depths, the microbial communities associated with WC and WW are distinct from one another. Separation associated with CV1 indicates the greatest differences exist between the WC and WW treatments. Both WW and SW-WW are positively correlated with CV1, whereas WC and SW-WC are negatively correlated. The discriminant functions associated with CV1 and CV2 accounted for 79 and 16% of the variance for a total explained variance of 96% at the 0–5 cm depth (Figure 2A). At the 5–10 cm depth, CV1 and CV2 accounted for 74 and 22%, combining for a total explained variance of 97% (Figure 2B). Because CV1 accounts for the bulk of the variance, the mean ellipse of WW at the 0–5 cm depth shows significant difference from those of WC and SW-WC, while the ellipses of WC and SW-WC indicate the greatest similarities (Figure 2A). Similarly, the greatest differences at the 5–10 cm depth are between WW and both WC and SW-WC.

The magnitude and positive correlation to both CV1 and CV2 in the surface soil suggest that T-PLFA has the greatest influence on the community associated with WW. The communities associated with SW-WW appear to be strongly influenced by fungi and AM fungi. At 5–10 cm, AM fungi appear to be largely responsible for the separation of SW-WW, while fungi and T-PLFA align with the community of WW. Differentiation between the microbial communities of WC, WW, SW-WC, and SW-WW based on the amounts and types of PLFAs was significant at *p* < 0.05. At the 0–5 and 5–10 cm depth, CV1 discerned WC vs. WW and SW-WC vs. SW-WW (Table 2). Canonical variate 2 discriminated WC vs. SW-WC, and WW vs. SW-WW.

**Table 2:** Structure matrix (pooled within canonical structure) and biomarker means (group centroid) for the WC-WW-SW cropping sequence at the 0–5 and 5–10 cm depths.

	0–5 cm		5–10 cm	
	CV 1	CV 2	CV 1	CV 2
Structure loading				
Fungi	0.51	−0.05	0.62	0.43
AM Fungi	0.72	0.13	0.69	0.13
Gram-	0.21	0.70	0.36	0.72
Gram+	0.26	0.27	0.22	0.49
Total	0.56	0.55	0.29	0.53
Group centriods				
WC	***−0.63***	0.30	***−0.30***	0.44
WW	***0.96***	0.32	***1.18***	0.38
SW-WC	***−0.77***	−0.13	***−1.30***	−0.02
SW-WW	***0.45***	−0.48	***0.40***	−0.79

Bold italics indicate positive and negative correlations to the canonical variates.

The absolute abundance of microbial lipid biomarkers is reported for each crop year at the 0–5 cm depth in Table 3. The observed pattern, though not always significant, indicates greater microbial biomass for all lipid groups and T-PLFA in WW vs. WC and SW-WW vs. SW-WC. Notable differences in abundance occur among AM fungi in the winter-crop cycle of the crop sequence where AM fungi are significantly greater in WW compared to WC in two of the 5 years with the other crop years following the same trend. Furthermore, abundance of AM fungi in the spring-crop cycle of the crop sequence was significantly greater in SW-WW vs. SW-WC in three of the five crop years (Table 3). Among crop years, the abundance of all microbial lipid groups in 2012 was significantly greater in WW vs. WC, apart from fungi. Additionally, the abundance of all microbial lipid groups in 2013 was significantly greater in WW and SW-WW treatments over WC and SW-WC except for fungi in WC vs. WW, and Gram- in SW-WC vs. SW-WW (Table 3).

**Table 3:** Absolute abundance (nmol/g) of microbial lipid groups at 0–5 cm depth from each crop year (2009–2014).

Crop year	Treatment	Fungi	AM fungi	Gram−	Gram+	Total
2009	WC	1.59 AB	0.41 A	3.04 A	4.01 A	16.14 A
	WW	1.83 A	0.52 A	2.27 A	4.16 A	15.47 A
	SW-WC	0.93C	***0.09*** B	0.50 B	1.61 B	8.93 B
	SW-WW	1.35 BC	***0.34*** A	0.66 B	1.82 B	11.29 B
2010	WC	1.08 AB	0.13 B	0.68 B	1.50 B	9.01 A
	WW	1.63 A	0.22 AB	0.80 B	1.79 B	10.09 A
	SW-WC	0.76 B	0.23 A	2.12 A	3.70 A	14.01 B
	SW-WW	1.39 AB	0.29 A	1.85 A	3.51 A	13.79 B
2012	WC	0.67 AB	***0.12*** BC	***0.72*** A	***1.49*** B	***8.43*** B
	WW	1.13 A	***0.41*** A	***1.13*** B	***1.98*** A	***20.70*** A
	SW-WC	0.41 B	***0.07*** C	0.50 B	1.08 B	7.25 B
	SW-WW	0.56 AB	***0.18*** B	0.66 B	1.13 B	8.16 B
2013	WC	1.10 B	***0.19*** B	***1.22*** B	***3.01*** B	***11.93*** B
	WW	2.10 AB	***0.54*** A	***2.43*** A	***5.07*** A	***18.11*** A
	SW-WC	***1.56*** B	***0.30*** B	1.75 AB	***3.54*** B	***14.24*** B
	SW-WW	***3.15*** A	***0.54*** A	2.14 A	***5.51*** A	***19.75*** A
2014	WC	***1.10*** B	0.28 A	2.73 A	5.04 A	16.87 A
	WW	***2.01*** A	0.35 A	3.01 A	5.32 A	18.96 A
	SW-WC	1.23 AB	0.23 A	1.02 B	3.52 B	12.18 B
	SW-WW	1.57 AB	0.34 A	1.29 B	3.63 B	13.62 B

Values are least square means of individual crop years (n = 24). Bold italics indicate significant difference between WC and WW, or SW-WC and SW-WW (p ≤ 0.05).

**Figure 3:**
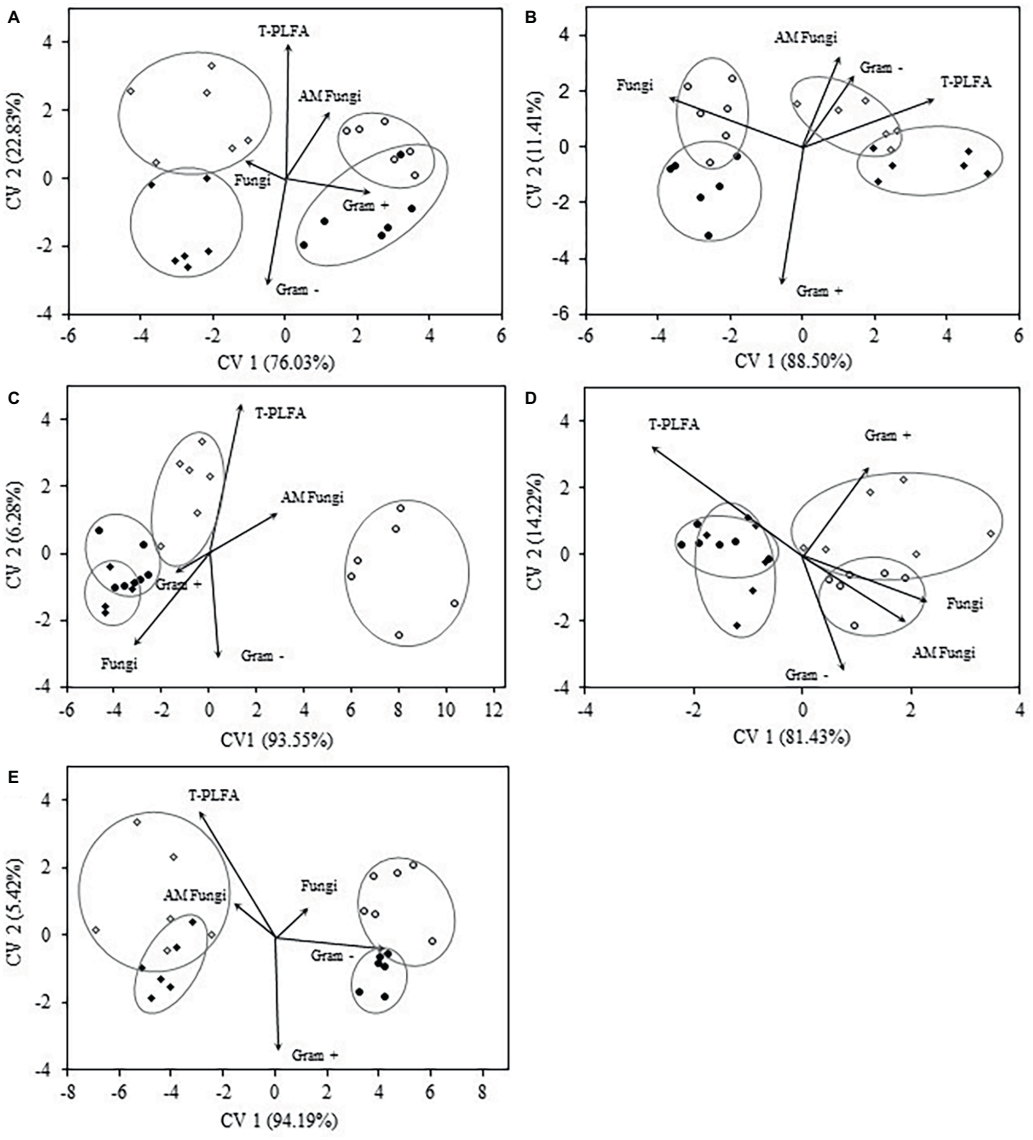
Canonical variates for lipid biomarker groups. Biomarker groups and total PLFA (T-PLFA) of the 0–5 cm depth for crop years 2009 **(A)**, 2010 **(B)**, 2012 **(C)**, 2013 **(D)**, and 2014 **(E)**. Vectors represent standardized canonical coefficients and indicate the contribution of each biomarker group to each canonical variate. Each sample point is represented and cluster by crop treatment. Each cluster is accompanied by a mean ellipse at the 95% confidence interval (treatments groups that differ significantly have confidence ellipses that do not intersect).

Discriminant analysis of microbial biomarkers for each crop year at the 0–5 cm depth is presented in Figure 3. In crop year 2009, the bi-plot reveals distinct separation of WC and WW treatments from SW-WC and SW-WW treatments along CV1 (76%). SW-WC and SW-WW separated along CV2 (23%), with mean ellipses sharing very little overlap (Figure 3A). The vectors for Gram+ and AM fungi were positively correlated with CV1, and total PLFA correlated with CV2. The community structure in 2010 showed significant differences between SW and the preceding crop (Figure 3B), with only small differences for WC vs. WW, and SW-WC vs. SW-WW. Compared to other crop years, 2010 was the only year where both SW-WC and SW-WW were positively correlated with CV1 (89%) and associated with the vectors of AM fungi, T-PLFA, and Gram− bacteria. For crop year 2012, the first CV (94%), discriminated WW from all other treatments, with SW-WW distinct from WC and SW-WC whose communities showed little differences. The vectors for T-PLFA and AM fungi correlated with both canonical variates (Figure 3C). The bi-plot for crop year 2013 discriminated WC and SW-WC from WW and SW-WW along CV1 (81%). Vectors of fungi, AM fungi, and Gram− all positively correlated with CV1, while the covariate for Gram+ is correlated with both CV1 and CV2 (Figure 3D). In crop year 2014, the community structure exhibited differences that discriminated WC and WW, from SW-WC and SW-WW along CV1 (94%). However, vectors for T-PLFA and AM fungi were negatively correlated, while fungi, and Gram− bacteria were positively associated with CV1 (Figure 3E).

## Discussion

Dehydrogenase enzymes aid in the oxidation of soil organic matter and are considered good indicators of microbial activity (Garcia-Gil et al., 2000). However, crop rotation treatments had little effect on dehydrogenase enzyme activity (DEA) of soil associated with WC compared to WW. Activity of dehydrogenase was significantly greater in WW vs. WC at the first sampling, but no consistent pattern was observed for the remainder of the experiment. While DEA has been reported to be a suitable indicator of microbial activity (Garcia-Gil et al., 2000), no differences in DEA associated with management practices have been reported (Kennedy and Schillinger, 2006; Pritchett et al., 2010). The intercellular nature of dehydrogenase and relation to the metabolic activity of soil microorganisms (Tabatabai and Dick, 2002) could explain why this enzyme assay may not be as responsive to immediate changes in management practices (Pritchett et al., 2010). In a similar study, differences in DEA were not observed until the third and fourth years of crop rotation and residue management (Schillinger et al., 2010).

Unlike DEA, crop rotation treatment did impact β-glucosidase activity. The rotation that included WW significantly increased BGA compared to WC. Because β-glucosidase enzymes are produced by bacteria and fungi, they are commonly found in agricultural soils (Shewale, 1981). Waldrop et al. (2000) showed a correlation between BGA and the microbial community composition of soils determined by PLFA. Hansen et al. (2018) reported that greater rhizosphere BGA in WW compared to WC was typically accompanied by greater abundance of total microbial biomass and individual biomarker groups in WW over WC. Knight and Dick (2004) previously reported correlations between microbial biomass and BGA. Therefore, reduced BGA suggests that WC led to reduced microbial biomass. This supports the results of 25.3 and 15.03% greater microbial biomass (T-PLFA) in WW vs. WC and SW-WW vs. SW-WC, respectively. While β-glucosidase enzymes are associated with bacteria and fungi, correlations between BGA and soil fungi have been reported (Hayano and Tubaki, 1985; Acosta-Martinez et al., 2007). Therefore, differences in BGA could correspond to soil fungal abundance which was significantly greater for WW vs. WC and SW-WW vs. SW-WC at both 0–5 and 5–10 cm depths.

While the results of this study indicate that fungal biomass contributes less to the total microbial biomass than bacteria, the influence of fungi is thought to be substantial. Among treatments, microbial lipid biomarkers indicate greater fungal abundance and AM fungi abundance in the WW compared to the WC treatments and supports the findings of Kirkegaard et al. (2000), who determined that high levels of GSL in the root tissues of canola grown in, or added to, soil are correlated with greater suppression of soil fungi. The differences in community structure between WC and WW treatments are well differentiated, indicating a shift in in the community from that which has developed under the historical rotations. The covariates of fungi, AM fungi, and T-PLFA are responsible for the discrimination in communities among treatments, with the covariates of bacteria having either a negative or null correlation with both canonical variates. Results of differentiated microbial communities agree with the research of Lay et al. (2018) who reported that the canola microbiome was significantly different from those of wheat and pea. Fungal populations in our study were significantly different, while the abundance of bacterial biomarkers was not significantly different but often lower in WC. This reduced influence on bacteria was also observed by Hu et al. (2015) who reported a decrease in fungal populations and a shift in the fungal community following additions of ITC, while less influence was observed in bacterial populations. Additionally, Smith and Kirkegaard (2002) determined that while ITC derived from canola roots negatively impacted both bacteria and fungi, bacteria were able to survive higher concentrations compared to fungi.

The microbial community of each individual crop year compared to the recorded yield data reveals some corresponding patterns in both abundance and community structure. These patterns could potentially explain the yield response of the subsequent SW crop. In crop years 2010 and 2013, SW yields were not significantly different (Schillinger and Paulitz, 2018), and in both cases, the community structure as well as the vector for AM fungi were positively correlated with CV1. The SW yield difference in 2010 was less than 2%, and there were no significant differences for any biomarker that year. However, biomarkers for 2013 were significantly different and may explain a yield difference of 9% of WW-SW over WC-SW.

Spring wheat yields in 2009, 2012, and 2014 were significantly reduced in SW-WC vs. SW-WW by 24.5, 28.1, and 37.9%, respectively (Schillinger and Paulitz, 2018). Averaged over the 5 years, highly significant differences in SW yield following WW vs. WC had *p* < 0.0001 (Schillinger and Paulitz, 2018). Microbial community structure for SW in 2009, 2012, and 2014 differentiates SW-WC from WW along CV1 and is negatively correlated with both CV1 and CV2. The only biomarker at the 0–5 cm depth significantly reduced in SW-WC for 2009 and 2012 was AM fungi and was significant in 2014 at *p* ≤ 0.10 (*p* = 0.0962). These three crop years also showed significantly reduced abundance for AM fungi in SW-WC at the 5–10 cm depth. In Northern Australia, reduced wheat yields in plots previously planted to canola were due to poor colonization with AM fungi (Owen et al., 2010), indicated by a significant correlation between yield of wheat and the levels of AM fungi following pre-crop treatments. In our study, 2011 had the highest overall WW yield and was the only year that WW was the only treatment positively correlated with CV1 (94%), with vectors for AM fungi and T-PLFA reflecting the largest contributions. Contrasting results from southern Australia indicate that AM fungi were not related to either positive or negative yield responses but rather residual N, water, and disease legacies (Kirkegaard and Ryan, 2014). The yield results reported by Schillinger and Paulitz (2018) were preceded by extensive analysis including residual N, P, S, and soil water, foliar and root diseases, weeds, and root-lesion nematodes. With these variables eliminated as the cause for the yield response, the results of Owen et al. (2010) appear to align with our findings.

Biomarkers identified as fungal consistently contained a measurable subset of mycorrhizal-related biomarkers. This suggests that our soil samples contained a resident population of mycorrhizae. If native populations of mycorrhizae exist, observed historical yields may be partially affected by mycorrhizal associations. Most crop species are considered mycorrhizal hosts in field situations and, therefore, possess a strategy that improves plant productivity (Smith et al., 2011). Under dryland conditions in the Pacific Northwest, native mycorrhizal colonization of wheat roots ranged from 13 to 26% (Mohammad et al., 1998) and increased to 51% with inoculation accompanied by a 25% increase in wheat grain yield. The findings presented here have confirmed reduced mycorrhizal biomarkers following canola. With reduced mycorrhizal abundance, the potential for colonization also declines. While that may be the case, contrasting reports argue that current wheat cultivars do not always form AM fungi relationships (Singh et al., 2012), modern breeding programs have reduced the responsiveness to AM fungi (Zhu et al., 2001), or response of subsequent crops was not related to AM fungal colonization (Kirkegaard and Ryan, 2014). However, our results presented here extend beyond the influence of AM fungi. Total PLFA and fungi across all years had significantly greater abundance in WW and SW-WW as well as positive correlation with the community structure.

Observed rotational effects of WC were limited to the subsequent SW crop. While the short-term effect was evident, the long-term influence was negligible. Differences in microbial biomass and microbial community composition as well as enzyme activities were not significant in the third year of the rotation, suggesting a recovery of soil biological conditions (data not shown). In 2 years of the 6-year study, SW was planted back-to-back (i.e., WC-SW–SW and WW-SW–SW) to determine the duration of the yield decline. No difference in SW yields was reported for these years (Schillinger and Paulitz, 2018), suggesting a short-lived rotation effect. Farmers in the region where this study was conducted have largely transitioned to planting spring canola in a WW-spring canola (SC)-NTF rotation. To date, farmers have observed no reduction in WW yield in the WW-SC-NTF compared with WW-SW-NFT rotation. Therefore, a 13-month fallow period following canola appears to resolve potential negative canola rotation effects.

## Conclusion

Crop rotation and diversification are generally recognized as management tools to enhance soil health in agroecosystems. A relevant objective for the use of rotation crops is to increase the performance of subsequent crops. The degree of influence on soil biological properties and crop productivity is, however, crop specific. Results showed that WC generally led to decreased microbial biomass compared to WW. Notably, fungi and AM fungi were more prone than bacteria to the apparent canola rotation effect. Declines in viable microbial biomass could interrupt key microbial processes related to nutrient and water acquisition essential for optimal crop productivity. While effects on the microbial community were observed within the initial WC and subsequent SW crops, the long-term effects (i.e., after 1 year) were negligible. Data from this study have helped regional farmers adjust their sequence of planting canola in wheat-based rotations that allow for continued crop diversification and to maintain optimum crop yield potential.

## Author Contributions

WS, TP, and JH designed the experiment. JH performed all laboratory analysis, statistical analysis, evaluated the data, and drafted the manuscript. JH, WS, TP, and TS all contributed to the final version of the manuscript.

### Conflict of Interest Statement

The authors declare that the research was conducted in the absence of any commercial or financial relationships that could be construed as a potential conflict of interest.
